# Transcriptomic Analysis of Viable but Non-Culturable *Escherichia coli* O157:H7 Formation Induced by Low Temperature

**DOI:** 10.3390/microorganisms7120634

**Published:** 2019-11-30

**Authors:** Junliang Zhong, Xihong Zhao

**Affiliations:** 1 Guangdong Province Key Laboratory for Green Processing of Natural Products and Product Safety, School of Food Science and Engineering, South China University of Technology, Guangzhou 510640, China; 2 Research Center for Environmental Ecology and Engineering, Key Laboratory for Green Chemical Process of Ministry of Education, Key Laboratory for Hubei Novel Reactor & Green Chemical Technology, School of Environmental Ecology and Biological Engineering, Wuhan Institute of Technology, Wuhan 430205, China

**Keywords:** viable but non-culturable, *Escherichia coli* O157:H7, RNA sequencing transcriptomic analysis, low temperature induction, formation mechanism

## Abstract

*Escherichia coli* O157:H7 is one of the most common pathogenic bacteria that pose a threat to food safety. The aim of this study was to investigate the mechanisms of the formation of viable but non-culturable (VBNC) *E. coli* O157:H7 induced by low temperature (−20 °C) using RNA sequencing (RNA-Seq) transcriptomics analysis. The results of the present investigation revealed the presence of 2298 differentially expressed genes in VBNC cells, accounting for 46.03% of the total number of genes. Additionally, GO function and KEGG pathway enrichment analysis were performed to investigate the functional and related metabolic pathways of the differentially expressed genes. We found that the ion transport, protein synthesis, and protein transmembrane transport activities were significantly improved in the VBNC cells, indicating that *E. coli* O157:H7 cells synthesized a considerable amount of protein to maintain the levels of their functional metabolic processes and life activities in the VBNC state. In conclusion, we suggest that the increased synthesis of proteins such as SecY, FtsY, and Ffh might indicate that they are the key proteins involved in the improvement of the transmembrane transport activities in VBNC *E. coli* O157:H7 cells, maintaining their functional metabolism in the VBNC state and enhancing their survival ability under low temperatures.

## 1. Introduction

*E. coli* O157:H7 is one of the most notorious foodborne pathogens known to cause gastroenteric infections with a low infectious dose and has often been isolated from raw fruits and vegetables, fresh cheese, raw milk, and undercooked beef and other meat products [1,2]. A series of food processing and storage activities such as drying, ultraviolet sterilization, pulsed light, low-temperature storage, addition of preservatives, etc., have the potential to induce the *E. coli* O157:H7 cells hidden in food ingredients into a viable but non-culturable (VBNC) state [3,4,5]. *E. coli* O157:H7 VBNC cells were induced by low temperature (8 ± 1.5 °C) for nine days on the surface of lettuce and spinach plants [6]. *Campylobacter jejuni* cells were observed to enter VBNC states by the incubation at 4 °C for 38 days [7]. Liu et al. [5] also found that VBNC *E. coli* O157 strains were acquired by the incubation in LB broth at −20 °C for 28 days. In our previous study, we confirmed that the *E. coli* O157:H7 detected in frozen beef balls was induced into the VBNC state after exposure to −20 °C for 152 days [8]. The low temperature-induced VBNC cells of *E. coli* O157:H7 pose a potential threat to the safety of frozen preserved food. Therefore, it is of substantial significance to investigate the related mechanism of the formation of VBNC *E. coli* O157:H7 induced by low temperature.

Although the mechanism of the induction of the VBNC state is not fully understood at present, several molecule proteins closely related to the formation mechanism have been investigated in depth. It was reported that the cold-induced VBNC cells of both *Vibrio vulnificus* [9] and *Staphylococcus aureus* [10] lost their catalase activity. Zhao et al. [4] reviewed the different views concerning the involvement of the RpoS (σS) protein in the induction of the VBNC state and established that this protein was the major stress regulator that enhanced the stress resistance and significantly affected the formation of VBNC cells. However, these previous studies only focused on a specific gene or protein, and have not well elucidated the mechanism of VBNC state induction. 

Transcriptome analysis technology can identify significant differentially expressed genes in VBNC cells, further revealing the related mechanisms of the VBNC state induction. Transcriptomic analysis was applied for the first time to the research of the formation mechanisms of VBNC *Vibrio cholera* cells, the results of which indicated that the upregulated protein synthesis and transport activities were beneficial to the maintenance of the survival of VBNC cells in artificial seawater at low temperatures [11]. In recent years, RNA sequencing (RNA-Seq) transcriptomics analysis has been used for the investigation of the mechanisms of the formation of various VBNC cells such as those of *E. coli* O157:H7 [12], *Lactobacillus acetotolerans* [13], *Rhodococcus biphenylivorans* [14], and *Brettanomyces bruxellensis* [15]. The findings reported in the above-cited literature show that the mechanism of the VBNC state induction varies depending on the influence of different environmental stress factors. In this study, to reveal the mechanism for the induction of the VBNC state, we employed RNA-Seq to analyze the differentially expressed genes in cold-induced VBNC *E. coli* O157:H7 and assess the metabolic pathways of VBNC cells at the transcriptional level. Finally, herein, we propose a putative mechanism of cold-induced VBNC cell formation, suggesting that the increased synthesis of transmembrane transport protein may be the key to the maintenance of the metabolic functions of VBNC cells and the VBNC state.

## 2. Materials and Methods

### 2.1. Bacterial Strains and Induction of VBNC E. coli O157:H7 by Low Temperature

*E. coli* O157:H7 ATCC 43895 obtained from the American Type Culture Collection (ATCC, Manassas, VA, USA) were grown on Difco™ Tryptic Soy Agar (TSA; Becton Dickinson and Co., Sparks, MD, USA) at 37 °C for 24 h and incubated overnight in Bacto™ Tryptic Soy Broth (TSB; Becton Dickinson and Co., Sparks, MD, USA) at 37 °C in a shaking bed (110 rpm). Following the procedure described in our previous study [8], non-culturable *E. coli* O157:H7 cells were generated in ground beef meatballs with 6.4 × 10^8^ CFU/mL culturable cells after exposure to −20 °C for 152 days. The direct viable count (DVC) assay, an identification method of live bacteria based on the substrate absorption ability, was used to determine whether these non-culturable cells were alive or dead [16]. This assay employed nalidixic acid as a DNA replication inhibitor, and the elongated cells can be judged as living cells. After the verification, over 10^7^ cells/mL of VBNC *E. coli* O157:H7 cells were harvested [8]. The samples in which the VBNC state had been previously confirmed were used for RNA-Seq analysis.

### 2.2. Preparation of RNA-Seq Samples

The cold-induced beef meatball samples were repeatedly centrifuged at 1000× *g* for 1 min to remove the ground beef, and VBNC cells at a concentration of approximately 10^7^ cells/mL were harvested for transcriptomic experiments. A total of 200 μL of overnight-incubated cell suspension was added to 10 mL of TSB and incubated at 37 °C for 2.5 h with 190 rpm shaking, obtaining logarithmic-phase (LP) cells, which were used at the same concentration as a control group. Three independent replicates were prepared for each condition described above.

### 2.3. RNA Extraction, Library Construction, and Sequencing

Total RNA of the *E. coli* O157:H7 sample was collected from three independent biological replicates for each condition. Then, using the Bacterial Total RNA Extraction kit (Sigma-Aldrich, Saint Louis, MI, USA), the samples were subjected to extraction according to the manufacturer’s instructions. The quality of the total RNA was determined by an Agilent 2100 Bioanalyzer (Agilent Technologies Inc., Santa Clara, CA, USA). The Ribo-zero™ rRNA kit (Epicenter Inc., Madison, WI, USA) was used to remove trace DNA and ribosomal RNAs. The mRNA enriched by the removal of ribosomal RNA was fragmented into short fragments of 200–700 bp using fragmentation buffer. Furthermore, the mRNA was reverse-transcribed into cDNA with 100 pmol of hexamer random primers by using the First Strand Master Mix (Invitrogen Inc., Carlsbad, CA, USA) and SuperScript III reverse transcriptase (Invitrogen Inc). Second-strand cDNAs were synthesized using the SuperScript Double-Stranded cDNA Synthesis kit (Invitrogen Inc.) in accordance with the instructions. A suitable size range of cDNA fragments were selected by agarose gel electrophoresis, followed by amplification by PCR. Finally, the sequence libraries with fragments with a length from 200–700 bp were constructed. The RNA-Seq library was evaluated using an Agilent 2100 Bioanalyzer, and the qualified sequencing library was sequenced on the Illumina HiSeq™ 2500 platform (Illumina, San Diego, CA, USA).

### 2.4. RNA-Seq Data Analysis

The RNA-Seq analysis in this study was performed by the Gene Denovo Biotechnology Company (Guangzhou, China). Using a base-calling procedure, the original image data obtained from the Illumina HiSeq™ 2500 platform were transformed into raw reads, which were stored in FASTQ format [17]. All raw data were deposited in the NCBI Sequence Read Archive (SRA) database and are accessible through the SRA Series accession number SRR6513355. After removing the raw reads containing adapters or low quality bases, the high-quality clean reads were mapped to the rRNA database by a short reads alignment tool, Bowtie2 [18], and the rRNA mapped reads were removed. The remaining clean reads were then mapped to the reference genome sequence of *E. coli* O157:H7 EDL933 (GenBank accession number NC_002655.2) by TopHat2 (version 2.0.3.12, The Center for Computational Biology of University of Maryland, MD, USA), and only mismatches with no more than two bases were allowed in the alignment [19]. Then, the randomness of mRNA/cDNA fragmentation was evaluated [20], and the gene coverage was calculated by the percentage of the covered gene. The gene expression levels of the LP and VBNC cells were calculated by using FPKM (fragments per kilobase of transcript per million mapped reads) [21]. The edgeR package (Available online: http://www.rproject.org/) was utilized to identify the differentially expressed genes between the two samples, and the genes with |log_2_ (fold change)| > 1 and false discovery rate (FDR) < 0.05 were considered significant differentially expressed genes. The significant differentially expressed genes were then subjected to enrichment analysis using the Gene Ontology (GO) functions and Kyoto Encyclopedia of Genes and Genomes (KEGG) pathways, which were performed to investigate the functional and related metabolic pathways of the differentially expressed genes. The RNA-Seq analysis was performed in independent triplicate biological experiments, and the final data presented in this manuscript were based on the mean values of the data provided by the Gene Denovo Biotechnology Company (Guangzhou, China).

### 2.5. Accession Number

All the raw data have been deposited in the NCBI Sequence Read Archive (SRA) database and are accessible through SRA Series accession number SRR6513355 (Available online: https://www.ncbi.nlm.nih.gov/sra/?term=SRR6513355).

## 3. Results

### 3.1. Comparison with the Ribosomal RNA Database

The results are shown in Table S1, where the numbers of the reads in the LP and VBNC cells unmatched with the data of the ribosome databases can be seen, which were 5,924,186 (82.58%) and 7,645,510 (90.42%), respectively. The next step was to compare the unmatched reads with the reference genome.

For the LP and VBNC cells, a total number of 5,647,634 (95.33%) and 6,983,517 (91.34%) reads respectively matched the reference genome (Table S2). Therefore, the transcriptional sequence data of the two groups were reliable and could be used for the subsequent sequencing analysis. 

### 3.2. Gene Expression Level in the Samples

The gene expression levels of the LP and VBNC cells were calculated by FPKM (fragments per kilobase of transcript per million mapped reads) [21], and the top five gene expression levels are presented in Table 1. The non-redundant (NR) database is one of the protein databases in NCBI, and the NR annotations of the five genes with the highest expression in the LP cells were those of glutamate decarboxylase β, translation inhibitory protein RaiA, cytidine phosphate PhoE, outer membrane protein A, and flagellin FliC. However, the NR annotations of the VBNC cells were those of the outer membrane protein A, DNA-directed RNA polymerase subunit α, elongation factor Tu, preprotein translocase subunit SecY, and 50S ribosomal protein L15. These results provided preliminarily evidence that differences existed between the VBNC and LP cells in the morphological characteristics of the cell membrane, protein translation processes, and transmembrane transport.

### 3.3. Statistics of the Differentially Expressed Gene between the Samples

**Figure 1:**
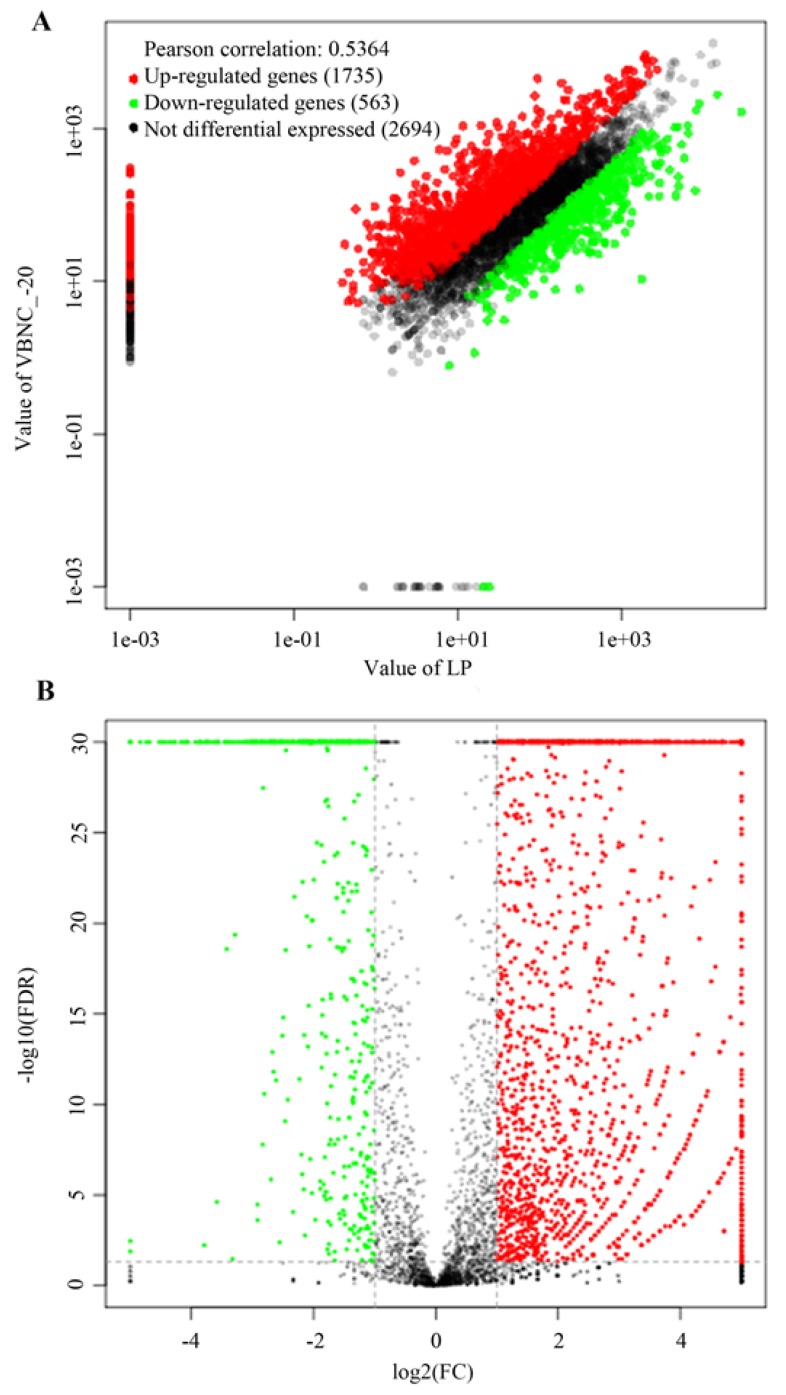
Differential expressed gene statistics between the VBNC and LP cells. (**A**) The scatter plot; (**B**) the volcano map.

The statistical results of the differentially expressed genes are presented by a scatter plot and a volcanic map, as illustrated in Figure 1. In the scatter plot (Figure 1A), the horizontal and vertical coordinates represent the expression of the LP and VBNC cells, the red (upregulated) and green (downregulated) spots denote the gene expression differences, whereas the black spot indicates the absence of differences. In the volcano map (Figure 1B), the abscissa represents the value of log2(FC), and the ordinate represents the negative log10 value of the FDR of the two samples.

As a result, a total of 2298 differentially expressed genes in VBNC cells including 1735 upregulated genes and 563 downregulated genes were obtained by comparing the gene expression levels of the cells in the LP and VBNC state, which accounted for 46.03% of the total number of genes. 

### 3.4. GO Functional Analysis

**Figure 2:**
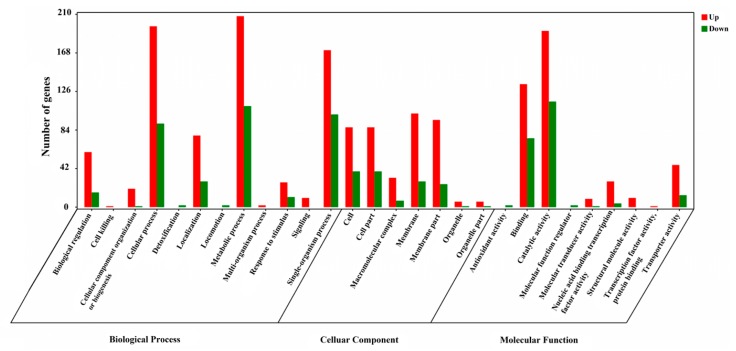
Significantly enriched GO terms of differentially expressed genes.

The functions of the differentially expressed genes obtained in the experiment were predicted and annotated by GO function analysis. The GO terms that were significantly enriched in the three GO analysis categories (Biological Process, Cellular Component, and Molecular Function) can be seen in Figure 2. As a result, “cellular process”, “metabolic process”, and “single-organism process” were significantly enriched in the category “Biological Process”, whereas “membrane” and “catalytic activity” were the most significantly enriched GO terms in the categories “Cellular Component” and “Molecular Function”, respectively. In each category of the GO ontology analysis, the GO term with the smaller *p*-value indicates that the differentially expressed genes in the term are more significantly enriched. The top three GO terms in every GO analysis category at a *p*-value of less than 0.05 are listed in Table 2. In Table 2, “ion transport”, “cell periphery”, and “organic anion transmembrane transporter activity” were significantly enriched in the “Biological Process”, “Cellular Component”, and “Molecular Function” categories, respectively.

The more repetitions, the differentially expressed genes in GO functional analysis are more significant. According to the statistics of differences expression genes in each GO term, the repetitive times of genes in the above-mentioned nine GO terms (Table 2) have been collected in Table 3, and the most significant differentially expressed genes in GO functional analysis were obtained including the genes *dcu*A, *pro*Y, *met*N, *glp*T, and *mgt*A. The five-repeated times were all in the categories of Biological Function and Molecular Function including GO:0006811, GO:0015711, GO:0008514, GO:0022891, and GO:0015075, which represented the annotation of “ion transport”, “organic anion transport”, “organic anion transmembrane”, “substrate-specific transmembrane transporter activity”, and “ion transmembrane transporter activity”, respectively.

### 3.5. KEGG Pathway Analysis

**Figure 3:**
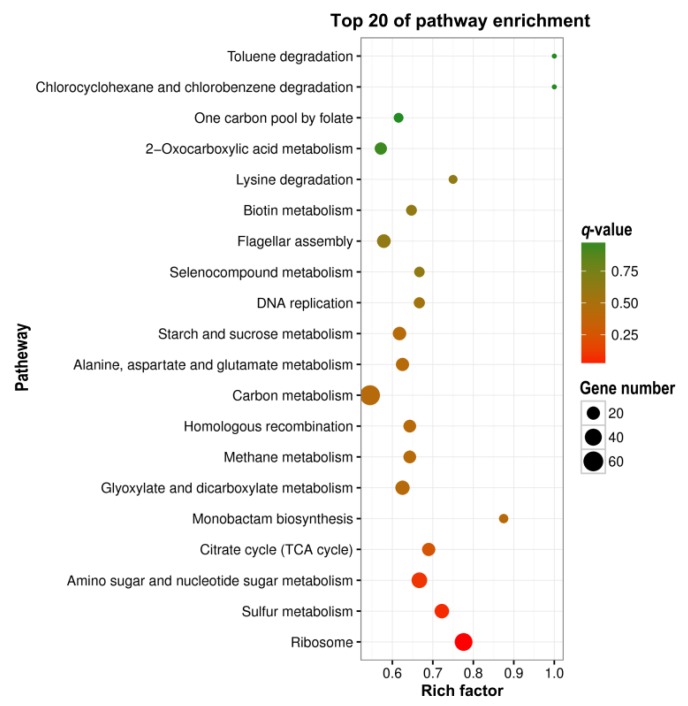
The top 20 of the pathway enrichment of differentially expressed genes between the VBNC and LP cells.

KEGG is the main public database of the pathway, and the major biochemical metabolic and signal transduction pathways of differentially expressed genes can be identified by KEGG pathway enrichment analysis [22]. In this study, the top 20 of the pathway functionally enriched differentially expressed genes between VBNC and LP cells were obtained (Figure 3), and the top five of the pathways, based on their *p*- and *q*-values from small to large, are presented in Table 4. Only the pathway of ID ko03010 (*q*-value = 0.000059) met the requirement of *q*-value ≤ 0.05, which represented the signal pathway of ribosome (ribosome pathway).

The differentially expressed genes of the ribosome signaling pathway obtained by the KEGG pathway analysis are listed in Table 5. The protein function annotations of the upregulated genes were consistent with those of RNA polymerase subunits α (RpoA) and β (RpoB), protein synthesis initiation factor IF (IF-1, IF-2, and IF-3), protein synthesis elongation factor EF (EF-Tu, EF-Ts, and EF-G), precursor protein translocase subunit SecY, and FtsY receptor and Ffh protein of the SRP transport pathway. Only the *rpm*E gene encoding for protein synthesis release factor RF-1 was significantly downregulated in the ribosome signaling pathway.

## 4. Discussion

**Figure 4:**
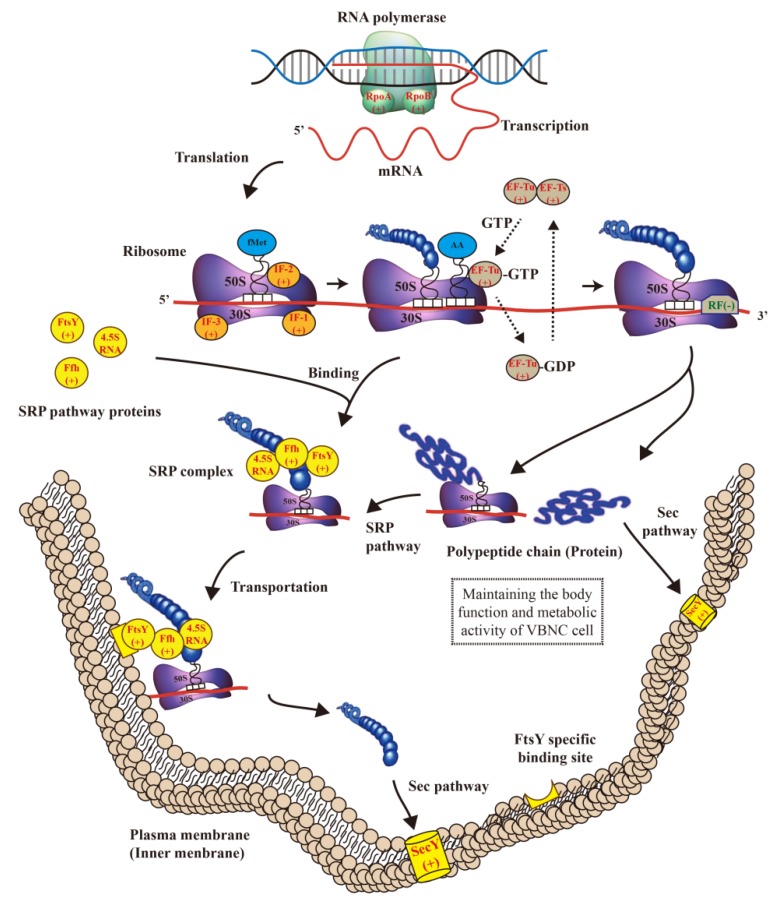
Metabolic pathway analysis of VBNC *E. coli* O157:H7 cells. (+) represents upregulated proteins, and (−) represents downregulated proteins.

Based on the results of the transcriptional data analysis, we assumed that the metabolic mechanism of the cold induction of the VBNC *E. coli* O157:H7 cells at the transcription level is associated with the processes of RNA transcription, protein synthesis, and protein transport in VBNC cells, which is illustrated in Figure 4. In previous studies, RNA polymerase was found to combine with the DNA template during the transcription process, moving from the 3′ end of the DNA template to the 5′ end after opening the double-strand DNA molecule [23]. The two subunits of RNA polymerase, RpoA and RpoB, were upregulated and accelerated the transcription of mRNA. When the ribosome recognized the promoter sequence on the 5′ end of mRNA, the initiation factor IF-3 promoted the separation of the large and small subunit in the ribosome, and IF-1 occupied the A-site on the small subunit, preventing its binding to other tRNA molecules [24]. The mRNA then bound to the small subunits, and IF-2 induced fMet-tRNA to integrate into the mRNA based on the principle of complementary pairing. Finally, a translation initiation complex was formed after the association of the large and small subunits [24]. Therefore, the upregulated expression of the initiation factor IF promotes the rapid formation of the protein translation initiation complex of VBNC cells during its formation. Under the action of the upregulated elongation factors EF-Tu and EF-Ts, amino acids continuously linked to peptide chains recognizing the respective codon on mRNA, and a GTP was transformed into GDP when an amino acid was connected. At the same time, the initial complex moved from the mRNA 5′ end to 3′ end as also established earlier [25]. The SRP pathway, a targeted transport pathway for newly synthesized protein, is constituted mainly by the signal recognition particle protein Ffh, the signal recognition particle-docking receptor protein FtsY, and 4.5S RNA [26]. In the process of peptide elongation, these SRP pathway proteins can recognize and combine with the signal peptide generated by the ribosome to form a SRP complex. However, if the ribosome releases the new peptide chain, the SRP pathway proteins cannot bind to the signal sequence [26]. The SRP complex carries the ribosome undergoing the process of translation to a FtsY specific binding site on the cell membrane, releasing a new peptide chain to pass through the SecY channel protein to complete transmembrane transportation [26]. The peptides involved in the SRP transport pathway may be related to the formation mechanism of VBNC cells. On the other hand, the downregulation of the release factor RF-1 leads to the formation of peptides containing the terminated codon UAA or UAG that cannot stop extending, which results in massive synthesis of peptides [27]. However, the release factor RF terminates the extension of some of the peptide chains. The newly-released peptide chains or proteins may maintain the body function and metabolic activity of the VBNC cell. Finally, the transmembrane transportation of peptide chains or proteins completed by the Sec transport pathway could maintain the metabolic balance of VBNC cells. In brief, the upregulation of RpoA and RpoB accelerated the transcription of mRNA in the transcription process. The upregulated IF factor promoted the rapid formation of the protein translation initiation complex of VBNC cells. The upregulated elongation factors EF-Tu and EF-Ts were important for the continuous linking of peptide chains. The downregulation of the release factor RF-1 resulted in massive synthesis of peptides. The upregulated proteins of SRP pathway and Sec pathway could satisfy the transportation needs of a large synthesizing of peptides and accelerate the protein transmembrane and targeted transportation.

As can be observed in Figure 4, the synthesis of the related proteins in our examination was upregulated to maintain the functional metabolic processes and life activities of the cold-induced VBNC *E. coli* O157:H7 cells. In comparison with the findings of previous research, Asakura et al. [11] found that the upregulated protein synthesis and transport activities were beneficial for the maintenance and survival of VBNC *V. cholera* cells at low temperature in artificial seawater. Our data have indicated that the cold-induced by −20 °C VBNC cells were significantly different from the LP cells and revealed that low temperature was one of the important factors at the gene expression level that led to the entry of *E. coli* O157:H7 into the VBNC state. Additionally, Lleò et al. [28] established that the upregulated EF-Tu and EF-Ts promoted the increased protein synthesis in VBNC *Enterococcus faecalis* cells. On the other hand, the response of bacteria to different environmental stress factors is different, and the intracellular metabolic mechanism of reaction of VBNC cells is also different. For example, Zhao et al. [12] detected the downregulated genes and proteins related to DNA replication, cell division, central metabolisms, and membrane transport were in VBNC *E. coli* O157:H7 induced by high-pressure CO_2_ (HPCD). These results suggest that the low metabolic activity could reduce the energy loss of the HPCD-induced VBNC cells and is beneficial to the survival of VBNC cells in the extreme environment of HPCD. Moreover, Capozzi et al. [15] revealed that the expression of the genes involved in the carbohydrate metabolism, amino-acid transport, and transporter activity was observed during the resuscitation of SO_2_-induced VBNC *B. bruxellensis* cells. The findings of these researchers strongly suggest that the formation mechanism of SO_2_-induced VBNC *B. bruxellensis* cells is associated with the response to oxidative stress and sulfite toxicity. 

Even an exceedingly low concentration of *E. coli* O157: H7 (e.g., only 10 cells) can cause infection and illness to humans. More importantly, VBNC *E. coli* O157:H7 cells are also a potential threat to food safety. VBNC cells of *E. coli* O157 [29] and *Salmonella* Oranienburg [30] were reported to have been likely to be involved in foodborne outbreaks. Therefore, it is extremely important to extend our knowledge of the metabolic activities and mechanism of formation of VBNC cells. Then, based on the formation mechanism revealed, methods and measures can be developed to prevent infections caused by VBNC cells. In this study, we investigated the mechanisms of the formation of VBNC *E. coli* O157:H7 induced by low temperature (−20 °C) by using RNA-Seq transcriptomics analysis. Lamas et al. [31] reported almost transcriptomic techniques applied on foodborne pathogens, and believed that transcriptomic research is of great importance to fully understand the resistance mechanisms and metabolic pathways involved in foodborne pathogens. We also believe this and used this transcriptomic tool to establish that the expression of the genes encoding for protein synthesis and transport in the VBNC cells were upregulated, especially those of the Sec and SRP transport pathways. Finally, we propose a putative formation mechanism of the cold-induced VBNC *E. coli* O157:H7 cells, suggesting that the increased protein synthesis and transmembrane transport activities observed in VBNC cells may enhance survival ability under low temperature and may be related to the formation of VBNC cells. Proteomic studies can be employed in our future experiments to investigate the novel proteins with increased expression in VBNC cells to obtain more extensive and deeper information about the mechanisms of the formation of hypothermia-induced VBNC cells.

## Figures and Tables

**Table 1:** The top five gene expression level in LP and VBNC cells sample.

Sample	Ranking	Gene	FPKM	Reads	NR Annotation
LP	1	Z_RS10405	29,197.3	64,534.96	Glutamate decarboxylase beta (*E. coli* O25b:H4-ST131) (*E. coli*)
	2	*rai*A	14,869.5	8023	Translation inhibitor protein RaiA (*E. coli* O157:H7 str. Sakai) (*E. coli*)
	3	*pho*E	14,297.2	24,902	Porin protein PhoE (*E. coli*)
	4	*omp*A	13,175.65	21,639	Outer membrane protein A (*E. coli* MS 107-1) (*E. coli*)
	5	*fli*C	12,644.53	35,070	flagellin FliC (*E. coli*)
VBNC	1	*omp*A	12,956.99	26,842	Outer membrane protein A (*E. coli* MS 107-1) (*E. coli*)
	2	*rpo*A	9133.91	17,995	DNA-directed RNA polymerase subunit alpha (*E. coli* O157:H7 str. Sakai) (*E. coli*)
	3	*tuf*	9112.96	21,490.06	Elongation factor Tu (*E. coli* CFT073) (*E. coli*)
	4	*sec*Y	8488.26	22,500	Preprotein translocase subunit SecY (*Shigella boydii*)
	5	Z_RS21965	8033.14	6954	50S ribosomal protein L15 (*E. coli* O157:H7 str. Sakai) (*E. coli*)

**Table 2:** The enriched GO terms of differentially expressed genes between VBNC cells and LP cells.

GO Category	GO Term ID	NR Annotation	GeneRatio	BgRatio	*p*-Value
Biological Process	GO:0006811	Ion transport	39 (8.44%)	58 (5.88%)	0.000997
	GO:0044282	Small molecule catabolic process	12 (2.6%)	14 (1.42%)	0.003079
	GO:0015711	Organic anion transport	20 (4.33%)	27 (2.74%)	0.003370
Cellular Component	GO:0071944	Cell periphery	9 (3.64%)	10 (2.03%)	0.010471
	GO:0005618	Cell wall	8 (3.24%)	9 (1.83%)	0.019240
	GO:0030312	External encapsulating structure	8 (3.24%)	9 (1.83%)	0.019240
Molecular Function	GO:0008514	Organic anion transmembrane transporter activity	14 (3.28%)	18 (1.92%)	0.005266
	GO:0022891	Substrate-specific transmembrane transporter activity	43 (10.07%)	71 (7.58%)	0.006012
	GO:0015075	Ion transmembrane transporter activity	28 (6.56%)	43 (4.59%)	0.006591

This table lists the top three GO terms in each GO analysis category, and the *p*-value is required to be less than 0.05.

**Table 3:** Repetitive differences expression genes in nine GO terms.

Number of Repetitive Times	Differences Expression Gene	NR Annotation
5	*dcu*A, Z_RS24500, Z_RS23645, Z_RS21565, Z_RS21275, Z_RS18545, *tyr*P, Z_RS12305, *pro*Y, *met*N, *glp*T	IT, OAT, OATTA, SSTTA, ITTA
4	*mgt*A	IT, OAT, SSTTA, ITTA
3	Z_RS26985, Z_RS26485, Z_RS25225, Z_RS24665, Z_RS22875, Z_RS22705, Z_RS22635, Z_RS22415, Z_RS21515, Z_RS17370, Z_RS17145, Z_RS13740, Z_RS09350, Z_RS09310, Z_RS06530, Z_RS05705, Z_RS02760, Z_RS02175, Z_RS24940, *nik*E, *msb*B, *kdp*C	IT, CP, CW, EES, SSTTA, ITTA
2	Z_RS22695, Z_RS16125, Z_RS13335, Z_RS12460, Z_RS10735, Z_RS03375, *ugp*C, *rht*B, *kdg*T, *brn*Q	IT, OAT, SSTTA, ITTA
1	Z_RS26780, Z_RS26335, Z_RS25895, Z_RS25370, Z_RS24525, Z_RS24370, Z_RS23610, Z_RS23465, Z_RS22905, Z_RS21350, Z_RS21150, Z_RS21140, Z_RS21025, Z_RS19885, Z_RS18625, Z_RS16005, Z_RS12610, Z_RS12490, Z_RS09475, Z_RS09120, Z_RS04060, Z_RS03890, Z_RS02910, Z_RS02490, Z_RS01960, *sec*F, *pst*S, *pst*A, *nep*I, *lam*B, *gld*A, *gcv*T, *gcv*H, *fuc*I, *fgh*A, *fad*E	IT, SMCP, SSTTA

“IT” = ion transport; “SMCP” = small molecule catabolic process; “OAT” = organic anion transport; “CP” = cell periphery; “CW” = cell wall; “EES” = external encapsulating structure; “OATTA” = organic anion transmembrane transporter activity; “SSTTA” = substrate-specific transmembrane transporter activity; “ITTA” = ion transmembrane transporter activity.

**Table 4:** The first five data of the pathway enrichment of differentially expressed genes between VBNC and LP cells.

Pathway ID	Pathway	DEGs Genes with Pathway Annotation (681)	All Genes with Pathway Annotation (1480)	*p*-Value	*q*-Value
ko03010	Ribosome	45 (6.61%)	58 (3.92%)	0.000001	0.000059
ko00920	Sulfur metabolism	26 (3.82%)	36 (2.43%)	0.001160	0.059179
ko00520	Amino sugar and nucleotide sugar metabolism	32 (4.7%)	48 (3.24%)	0.002740	0.093167
ko00020	Citrate cycle (TCA cycle)	20 (2.94%)	29 (1.96%)	0.010074	0.256884
ko00261	Monobactam biosynthesis	7 (1.03%)	8 (0.54%)	0.020602	0.420276

**Table 5:** Differential expression genes of ribosomal signaling pathway in the pathway functional analysis.

**Pathway ID**	**Signal Pathway**	**Differentially Expressed Genes in the Signal Pathway**
Ko03010	Ribosome	*rps*O, *rpl*S, *rps*M, *rpl*F, Z_RS23800, *rpl*T, *rps*Q, *rps*R, *rps*D, *rps*B, *rpl*U, Z_RS08020, *rpl*B, *rpl*R, *rpl*D, *rpl*V, Z_RS16205, *rps*C, *rpl*C, *rpl*W, *rpmC*, Z_RS25730, *rpl*X, Z_RS25530, *rps*I, *rpl*M, *rps*E, *rpm*A, *rpl*P, *rps*K, *rpm*G, *rpl*E, *rpmI*, *rps*G, *rps*S, *rpm*D, Z_RS20780, *rps*J, Z_RS27210, *rpl*J, *rpl*Q, Z_RS27195, *rpl*O, Z_RS00125, Z_RS30885
**Related protein annotations**		**Differentially expressed genes of significant protein annotation in the signal pathway**
RNA Polymerase Subunit α and β (+)		Subunit α: *rpl*Q, *rpl*M, *rps*I; Subunit β: *rpl*J
Initiation factor (+)		IF-1: *rps*M, *rps*K, *rps*D; IF-2: *rps*O; IF3: *rpm*I, *rpl*T
Elongation factor Tu (+)		EF-Tu: *rps*J, *rpl*C, *rpl*D, *rpl*W, *rpl*B, *rps*S, *rpl*V, *rps*C, *rpl*P, *rpm*C; EF-Ts: *rps*B; EF-G: *rps*G
Preprotein Translocase Subunit SecY (+)		*rps*Q, *rpl*X, *rpl*E, *rpl*F, *rpl*R, *rps*E, *rpm*D, *rpl*O
FtsY and Ffh (+)		*rpl*S
release factor RF-1 (−)		*rpm*E

“+” = upregulated; “−” = downregulated.

## Supplementary Materials

The following are available online at https://www.mdpi.com/2076-2607/7/12/634/s1.
