# Supplementary material for: Transcriptomic Analysis of Viable but Non-Culturable Escherichia coli O157:H7 Formation Induced by Low Temperature
Source: Microorganisms. 2019 Nov 30;7(12):634. doi: 10.3390/microorganisms7120634 (PMC6955965; doi:10.3390/microorganisms7120634)
Supplement: Supplementary file 1 [file microorganisms-07-00634-s001.pdf]

Supplementary Materials

Table 1. Comparative statistics between high quality reads and ribosomal databases.

Sample	All Reads Num	Mapped Reads	Unmapped Reads
LP	7174076	1249890(17.42%)	5924186(82.58%)
VBNC_-20	8455328	809818(9.58%)	7645510(90.42%)

Table 2. Comparative statistics between reference genomes and reads unmatched with rRNA.

Sample	Total Reads	Unmapped Reads	Unique Mapped Reads	Multiple Mapped reads	Mapping Ratio
LP	5924186	276552 (4.67%)	5605248 (94.62%)	42386 (0.72%)	95.33%
VBNC_-20	7645510	661993 (8.66%)	6908525 (90.36%)	74992 (0.98%)	91.34%
